# Relationship between Alcohol Consumption and Drinking Refusal Self-Efficacy among University Students: The Roles of Sports Type and Gender

**DOI:** 10.3390/ijerph17124251

**Published:** 2020-06-14

**Authors:** Youngho Kim, Garry Kuan

**Affiliations:** 1 Department of Sport Science, Seoul National University of Science and Technology, Seoul 01811, Korea; yk01@seoultech.ac.kr; 2 Exercise and Sports Science Programme, School of Health Sciences, Universiti Sains Malaysia, Kubang Kerian 16150, Kelantan, Malaysia; 3 Department of Life Sciences, Brunel University, London UB8 3 PH, UK

**Keywords:** drinking behaviour, drinking refusal self-efficacy, university students

## Abstract

The present study examines the relationship between drinking behaviour and drinking refusal self-efficacy among university students, with a focus on the influential roles of sports type and gender. A total of 354 students from Seoul National University of Science and Technology volunteered and participated in this study. The Korean Alcohol Use Disorders Identification Test (AUDIT-K), the Korean Drinking Refusal Self-Efficacy (DRSE) Questionnaire-Revised, and a questionnaire measuring participation in sports activities were administered to measure students’ alcohol drinking behaviours, refusal self-efficacy and sport-related activities, respectively. The data were analysed using descriptive statistics, two-way ANOVAs, correlation analysis, and regression analysis. The subdomains of DRSE had significant influences on binge drinking. Specifically, low DRSE in situations involving social pressure played a key role in predicting increased binge drinking. There was also a significant relationship between sports type and drinking behaviour, but only for male students. Those who participated in individual sports binge drank less than those who participated in team sports. Finally, gender differences were also revealed in drinking behaviour. Male students were more likely to binge drink compared to their female counterparts. This study offers a foundation for practical interventions to help control problematic drinking behaviour among university students.

## 1. Introduction

Alcohol is the most readily available and broadly used intoxicant, and excessive drinking is a major cause of health problems around the world [1]. Binge drinking is defined as the consumption of an excessive amount of alcohol in a short period of time (i.e., consume five or more alcoholic drinks in one sitting). In particular, binge drinking has gained much attention [2,3], as individuals who binge drink are more likely to engage in dangerous behaviour as compared to those who do not [4]. Although drinking-related problems occur across age groups, university students have the highest alcohol usage rate as well as the greatest frequency of binge drinking [1]. A report from 2017 revealed that 69% of college students in the United States (USA) reported using alcohol “in the past month,” and 29% were classified as binge drinkers [5]. Furthermore, a nation-wide survey indicated that 66% of university students had tried alcohol, and 46% reported binge drinking at some point [6]. Drinking rates are similarly high among Korean university students. According to the Korean Alcohol Research Foundation [7], 85% of Korean university students drank in the month prior to the survey. Moreover, 71.3% of the participants regularly consumed four or five alcoholic drinks in one sitting, with 42% being characterised as binge drinkers and 29% as extreme binge drinkers (i.e., consuming 10 or more alcoholic drinks in one sitting). Most Korean students (97% of the 5865 students surveyed) report having tried alcohol, with 91% reporting drinking in the previous month, and 52% reporting binge drinking [8].

In addition, it has been shown that university students’ drinking behaviour varies with gender [9,10]. Wagoner and colleagues [11] indicated that male students drink more frequently and heavily than their female counterparts. Although males are more likely to binge drink, females’ alcohol drinking behaviour is rising, and the gender gap in binge drinking is closing [12,13]. In the US, binge drinking rates have declined in the adult population as a whole while remaining steady for male students; however, binge drinking rates have increased by over 40% for female peers [13]. The fact that a sizable proportion of the university student population, both male and female, engages in routine binge drinking may result in acute and chronic health problems, involvement in various accidents, and anti-social behaviour [14,15]. For this reason, further research on binge drinking among university students should be a key priority.

The alcohol expectancy theory suggests that drinking behaviour is caused by individual beliefs and expectations regarding the effects of alcohol [16]. Furthermore, drinking refusal self-efficacy (DRSE), based on Bandura’s concept of self-efficacy [17], suggests that an individual’s belief in his or her ability to refuse alcohol in certain situations is a key factor underlying individual judgment and actual drinking behaviour [18]. Recently, Jongenelis et al. [19] indicated that individuals who engage in binge drinking have high alcohol expectancy and low DRSE. They seemed to have high expectations for positive outcomes when engaged in binge drinking. Oei et al. [20] identified three motivational factors for drinking, which contributed to DRSE: (1) opportunistic drinking, (2) social pressure, and (3) emotional relief. Previous studies have suggested that both alcohol expectancy and DRSE are important predictors of alcohol drinking behaviour with alcohol [21], and both are motivational factors that highlight the importance of individual beliefs. Within the framework of these two concepts, it is broadly accepted that people can encounter various situations or motivations for drinking.

Considerable research has been conducted on the relationship between the above-mentioned sub-factors of DRSE and drinking behaviour [20,22]. For instance, Oei and Jardin [21] reported that the overall DRSE was a significant predictor of alcohol-related behaviour, and in their study, Asian students had higher scores on measures of social pressure as compared to their Caucasian peers. Ding et al. [23] found that social pressure and perceived social norms regarding close friends’ drinking behaviour were associated with binge drinking. Similarly, the Korean Alcohol Research Foundation [7] found that university students are often unable to refuse alcohol when drinking with professors or senior students, and they argued that this type of situation can be recognised as social pressure. Moreover, Ehret et al. [24] found that DRSE was significantly associated with typical weekly drinking and negative consequences related to alcohol consumption. Participants who reported lower DRSE in the social pressure and emotion regulation dimensions were at greatest risk for heavy drinking and its related consequences.

Another interesting component of drinking behaviour is the fact that drinking is associated with one’s level of physical activity and sports participation [25,26]. Dinger et al. [27] reported that university students who engage in the recommended levels of physical activity have a lower tendency to engage in binge drinking. Moreover, higher levels of participation in sports activities by university students were found to be associated with a lower likelihood of alcohol abuse; in particular, students who participated in fitness-based physical activity had lower levels of drinking frequency and consumption [28]. Interestingly, alcohol consumption among university students is differentially associated with the type of sport participation [29,30]. For instance, Martens et al. [30] reported that university students participating in individual sports (i.e., swimming and diving) consume more alcohol than those who play team sports (i.e., soccer and volleyball). In contrast, some studies revealed that university students involved with team sports reported higher alcohol consumption than those competing in individual sports [31,32]. In sum, while several studies have investigated the influence of sports type on drinking behaviour, results have been inconsistent, and explanations for these differences are lacking.

Limited research has been conducted on the relationships between DRSE, sports type, and drinking behaviour among university students. Furthermore, the majority of studies have been conducted in Western countries. Research on this issue is lacking across Eastern countries such as Korea, where drinking behaviour and related DRSE have become a concern among young adults. Previous Korean studies have mainly dealt with prevalence, frequency, and amount of drinking reported by students as opposed to assessments of related factors such as perceived confidence in refusing drinks and sports participation. Therefore, the present study investigates the relationship between drinking behaviour and DRSE among Korean university students, with a particular focus on the influential role of sport type and gender.

## 2. Materials and Methods

### 2.1. Participants

A total of 354 university students (191 males, 163 females; *M*_age_ = 20.90 years, *SD* = 1.36) from Seoul Science and Technology (Seoultech) voluntarily participated in this study. The participants included 231 students who were involved with sports with the common sports activities being basketball, baseball, soccer, swimming, and yoga. However, the remaining did not participate in any physical activity. Seoultech is a public university located in Nowon-gu, Seoul, South Korea. It is the fifth-largest campus in the universities of Seoul and was ranked second among the public universities in 2018. Seoultech houses six colleges, 23 departments and seven graduate schools, with the enrolment rate of 11,500 students annually. Sample size calculation was conducted using G*Power 3.1.7 (Heinrich-Heine-Universität Düsseldorf, Dusseldorf, Germany). The expected correlation between binge drinking and DRSEQ’s subscales was 0.20 [18]. With the alpha value of 0.05, the power of 0.95, the estimated sample size was 319. After adding 10% of the dropout rate, a sample of 351 participants was judged to be sufficient for this study.

### 2.2. Data Collection

The study was approved by the Institutional Review Board of Seoul National University of Science and Technology (U2020-0740) and conducted in accordance with the Declaration of Helsinki using a cross-sectional study design. Participants were recruited via (a) a press release issued through the university, (b) recruitment flyers posted on university websites, and (c) an announcement made through classes with information about the study in January 2020. Recruitment occurred over one and a half months, and those who were interested in the study during this time period were added to a wait-list database. Inclusion criteria included fluency in the Korean language and being 18 years of age or older. Informed consent forms were given out before the study began. A total of 400 students gave their consent to participate. Of those, 354 students (88.50%) completed paper-and-pencil surveys. The remaining 40 (11.50%) were disqualified because they failed to complete the consent and survey forms. The estimated time to complete the survey was 10–20 min.

### 2.3. Instruments

Korean Alcohol Use Disorders Identification Test (AUDIT-K). In order to assess students’ binge drinking, two items were taken from the AUDIT-K developed by Kim and colleagues [33]. The first item was worded as “On how many days in the past two-weeks did you drink more than five glasses of alcohol within two hours?” A second question was worded as “On how many days in the past month did you drink at least one glass of alcohol?” For each question, respondents answered on a 7-point Likert scale as follows: 0 days (0), 1−2 days (1), 3−4 days (2), 5−6 days (3), 7−8 days (4), 9−10 days (5), and more than 11 days (6).

Drinking Refusal Self-Efficacy Questionnaire-Revised (DRSEQ-R). DRSEQ-R was originally developed by Oei and colleagues (2005) and was translated into Korean [34]. It was used to measure university student’s self-efficacy regarding refusal to drink in different situations. The DRSEQ-R comprised 19 questions, with 7 questions relating to the opportunistic drinking (e.g., “when I am at lunch”), 5 questions relating to the social pressure (e.g., “when someone offers me a drink”), and 7 relating to the emotional relief (e.g., “when I feel frustrated or helpless”). The answers were scored on a 6-point Likert scale from “I cannot stop myself at all (1)” to “I can definitely stop myself (6).” Initially, an exploratory factor analysis with Varimax rotation was performed to assess factors of the revised questionnaire. Factors with eigenvalues over 1 were considered important, and items were included with loadings of at least 0.50. The three factors (subscales) extracted were emotional relief (e.g., “when I am angry”), opportunistic drinking (e.g., “when I am listening to music or reading”), and social pressure (e.g., “when my friends are drinking”). Throughout these steps, three items with factor loadings below 0.40 were deleted from the original questionnaire, and a total of 16 items across the three subscales were finally applied to assess DRSE in this study. Table 1 shows the results of the exploratory factor analysis of 200 students. Cronbach’s coefficient α was as follows: 0.96 for emotional relief, 0.87 for opportunistic drinking, and 0.90 for social pressure.

Participation in Sports Activities. In order to assess students’ participation in sports activities, the following question was used: “Have you participated in a regular sports activity, at least 3 times per week, in the last 2 months? If so, please write down the name of the activity.” The answers were then classified into non-participation, team sports, and individual sports.

### 2.4. Statistical Analysis

All analyses were conducted using SPSS Version 25.0 (IBM, Armonk, NY, USA), and statistical significance was set at α = 0.05. Descriptive statistics were used to compare participants’ basic information, and an exploratory factor analysis was conducted on the DRSEQ-R. In order to assess the relationship between binge drinking and DRSE, Pearson correlations were analysed. A multiple linear regression analysis was employed to investigate the influence of DRSE subdomains (factors) on binge drinking. A two-way ANOVA (gender (2) × sport type (3)) was conducted to assess any effects of gender and sport type on DRSE and binge drinking. Before running the ANOVA and multiple linear regression analyses, normal distribution and homogeneity of variance assumptions were tested.

## 3. Results

### 3.1. Participant Characteristics

Table 2 shows the participants’ general characteristics. Regarding sports activities, 172 university students (48.59%) participated in team sports (e.g., basketball, tennis, baseball, soccer), 59 (16.66%) in individual sports (e.g., jogging, work-out, swimming, and yoga), and 123 (34.75%) did not participate in any physical activity. The mean score for binge drinking was 3.16, equivalent to binge drinking on five or six days during the previous two weeks. On average, participants drank at least one glass of alcohol on 12−13 days during the previous month (mean score = 3.39).

### 3.2. Binge Drinking According to Gender and Sport Type

**Figure 1:**
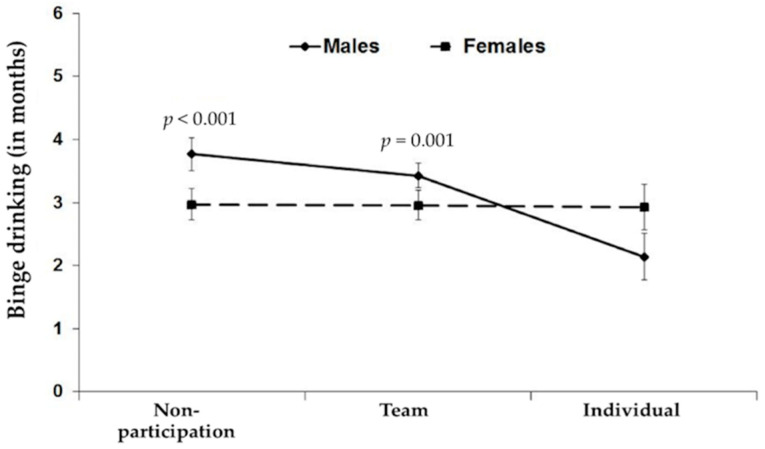
Effects of gender and sport type on binge drinking.

An interaction between gender and sport type was observed, *F*(2, 348) = 3.31, *p* < 0.05; with a main effect of sport type, *F*(2, 348) = 3.59, *p* < 0.05, but no main effect of gender, *F*(2, 348) = 3.31, *p* > 0.05 (see Figure 1). In order to further assess the interaction, simple main effects were tested for each independent variable. Results revealed that male students who did not participate in any sport tended to binge drink alcohol more often than male students who participated in individual sports, *t*(88) = 3.87, *p* < 0.001, and male students participating in team sports tended to binge-drink more frequently than those who participated in individual sports, *t*(58.90) = 3.54, *p* = 0.001. In contrast, female students’ binge drinking behavior did not differ as a function of sports type.

### 3.3. DRSE According to Gender and Sports Type

**Figure 2:**
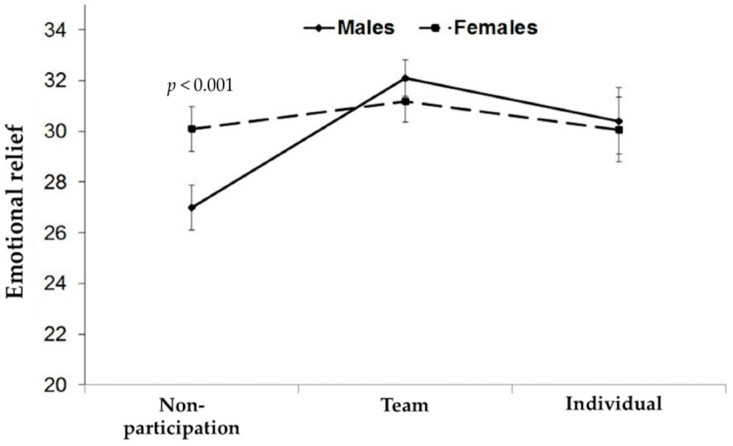
Effects of gender and sport type on emotional relief in the Korean Drinking Refusal Self-Efficacy (DRSE) questionnaire.

There was a significant interaction between gender and sports type on the emotional relief of the DRSE subdomains, *F*(2, 348) = 3.06, *p* < 0.05. Moreover, a main effect of sport type was observed, *F*(2, 348) = 6.98, *p* < 0.01 (see Figure 2). When posthoc analyses were conducted to further investigate the interaction, male students who did not participate in sports had lower DRSE scores during situations involving emotional relief compared to those participating in team sports, *t*(96.09) = −4.54, *p* < 0.001. Conversely, for female students, sports-type did not influence DRSE scores during situations involving emotional relief. There were no significant effects of sports type or gender on opportunistic drinking, *F*(2, 348) = 2.20, *p* > 0.05 and social pressure, *F*(2, 348) = 0.01, *p* > 0.05, in DRSE.

### 3.4. Relationship between Binge Drinking and DRSE

Table 3 shows the correlations between binge drinking and DRSE. All three DRSE subdomains were negatively and significantly correlated with binge drinking, with the highest correlation between social pressure and binge drinking (r = −0.45).

Table 4 shows the results of the multiple regression analysis examining the effect of DRSE subdomains on binge drinking. As shown in Table 4, social pressure (β = −0.42) and opportunistic drinking (β = −0.20) had a significant impact on male students’ drinking (*R*^2^ = 0.23). Meanwhile, only social pressure had a significant impact on alcohol drinking among their female counterparts (*β* = −0.40, *R^2^* = 0.16).

## 4. Discussion

Since problematic alcohol drinking habits acquired during university years can lead to unhealthy habits later in adulthood, assessing drinking behaviour among young adults is an important step for preventing harmful health-related behaviour in the future [3,7]. Therefore, the present study investigated the relationship between drinking and refusal self-efficacy (i.e., emotional relief, opportunistic drinking, and social pressure) among university students. Moreover, the study examined the effects of gender and sport type (i.e., team sports, individual sports, and no participation) on drinking behaviour and refusal self-efficacy.

Our retrospective study revealed that DRSE subdomains had negative influences on binge drinking. In particular, low DRSE in social pressure situations played a key role in predicting increased binge drinking. This result did not differ between male and female students. For male students with low DRSE, when there is an opportunity to drink, the social pressure factor will influence the students to binge drinking. These findings are similar to previous research showing that lower DRSE is associated with higher alcohol consumption [21,22]. Specifically, one Korean study revealed that social pressure was highly correlated with alcohol dependence when compared to the other DRSE subdomains [23]. This indicates that Korean university students are likely to binge drink when DRSE is reduced in situations where there is high social pressure [7]. Moreover, male students, who are more commonly exposed to alcohol than their female counterparts, may have difficulties achieving high DRSE and avoiding binge drinking in these types of social situations [35].

In a study comparing cognitive patterns related to DRSE among Asian and Caucasian university students [21], social pressure was an important factor for predicting binge drinking for Caucasian but not Asian students. Conversely, social pressure in the current study was the best predictor of binge drinking among our Korean university students. This discrepancy between studies could be due to sample characteristics. For instance, the current sample included Korean students attending university in Korea rather than in a different country (i.e., Oei and Jardim’s [21] Asian samples, including Koreans, were attending university in a Western country). Thus, participants in the present study could have been influenced by social pressures within their own familiar cultural context. In other words, this finding can be interpreted by considering that social motives are the most frequently endorsed reasons for alcohol drinking among university students [36], and collegiate alcohol drinking occurs primarily in social contexts in which overt or implicit peer pressure influences consumption [37].

Results from the current study indicated a significant relationship between sports type and drinking behaviour, but only for male students. Specifically, male students participating in individual sports binge drank less than those participating in team sports. Kim and Lee [38] reported differences in the amount of alcohol consumed based on sports type, demonstrating that university students participating in team sports consumed more alcohol than those participating in individual sports. It is plausible that socialisation processes within team sports could account for the effects of sports type on binge drinking observed in the present study. Owing to the nature of team sports, participants tend to form tighter-knit groups and are more outgoing [31]. In highly cohesive groups, a modelling effect (i.e., peer pressure, raising conformity), that is, members of a group copying each other’s behaviour, may appear in the form of drinking behaviour [31,33]. Relatedly, team sports involve more social contact and communication between participants compared to individual sports, which may lead to a greater opportunity for drinking and which may combine with social pressure to result in a greater likelihood of binge drinking.

The current findings also revealed that male students were more likely to binge drink compared to their female counterparts. The significant gender differences in binge drinking could be associated with differentiated drinking norms [39]. Although Korean society has changed, certain sectors tend to be more traditional. For men, drinking alcohol is a social custom that contributes to a shared group identity and eases conflict. Conversely, drinking for women is still considered socially unacceptable, although an increasing number of women are drinking [40]. Therefore, it might be that female students generally use protective strategies to drink less, and have higher expectations regarding self-control as compared to their male peers.

As with binge drinking, DRSE in situations involving emotional relief was also higher among students participating in sports than those who did not. In particular, this difference was significant for individuals engaging in team sports and was more prominent among male students. Engaging in sports is associated with diminished anxiety, depression, and stress and an increased sense of one’s own self-concept, self-esteem, and self-efficacy [41,42,43]. Since regular participation in sports and group cohesion within team sports could relieve negative psychological outcomes, there is less of a need to drink for emotional relief. Therefore, team sports participants with ample socialising opportunities should have high DRSE for situations involving emotional relief. This could not be substantiated from the literature, and there is still a need for further investigations to see whether it is an aspect of Korean society because the influence of sport type on drinking behaviour has been inconsistent in previous studies [30,31]. As with Lee and Choi [37], who found no difference in DRSE according to gender despite gender differences in alcohol consumption and binge drinking, no gender-based difference was noted in the present study for DRSE. In other words, university students with high DRSE binge drink less, regardless of gender. However, in order to further assess gender differences in DRSE, motivation for drinking and sports participation between men and women should be investigated.

The present findings add to the literature emphasising the importance of DRSE in understanding university students’ drinking behaviour. However, several limitations should be addressed in future research. A cross-sectional research design was employed in the present study; thus, caution must be taken with inferring causal relationships. Longitudinal studies are necessary to determine the causative role of self-efficacy on drinking. In addition, while the measures used underwent a rigorous and systematic translation and validation process, all measures were self-reported, which could lead to biases in interpretation or social desirability. Finally, as the primary focus of this study was to explore drinking behaviour among Korean university students and identify the possible relationship between drinking behaviour and DRSE, and interactions between DRSE and other demographic factors were not examined.

## 5. Conclusions

Results show that DRSE subdomains have negative influences on binge drinking; low DRSE in social pressure situations played a key role in predicting increased binge drinking. The ideas and issues identified are partially consistent with results from previous Western studies in the same field. Male students were more likely to binge drink compared to their female counterparts. Therefore, the present study offers a starting point for practical interventions to help control problematic drinking behaviour among university students.

## Figures and Tables

**Table 1:** Factor analysis for Drinking Refusal Self-Efficacy Questionnaire-Revised (DRSEQ-R).

Item	Factor 1	Factor 2	Factor 3
Q9 When I feel Down	0.92		
Q8 When I feel Upset	0.92		
Q12 When I feel Sad	0.86		
Q7 When I am Worried	0.83		
Q3 When I am Angry	0.78		
Q6 When I feel Frustrated	0.78		
Q2 When I am Watching TV		0.83	
Q14 When I am Listening to Music or Reading		0.78	
Q16 When I am by Myself		0.77	
Q19 When I First Arrive Home		0.75	
Q5 When I am at Lunch		0.74	
Q17 When I have Just Finished Playing Sport		0.62	
Q15 When my Friends are Drinking			0.91
Q18 When I am at a Pub or Club			0.86
Q4 When Someone Offers me a Drink			0.85
Q13 When my Partner is Drinking			0.77
Eigenvalue	9.05	2.89	1.88
% of Variance	47.62	15.19	9.90
Cumulative %	47.62	62.81	72.71
Cronbach’α	0.96	0.87	0.90

Factor 1 = emotional relief, Factor 2 = opportunistic drinking, Factor 3 = social pressure.

**Table 2:** General characteristics of the participants.

Characteristics		*n*	%
Gender	Male	191	54.0
	Female	163	46.0
Sport Type	Team sport	172	48.59
	Individual sport	59	16.66
	Non-participation	123	34.75
Binge Drinking (*M* ± *SD*)	3.16 ± 2.02		
Drinking Days (*M* ± *SD*)	3.39 ± 1.28		

**Table 3:** Correlation between binge drinking and DRSEQ-R.

DRSEQ-R			
	Emotional Relief	Opportunistic Drinking	Social Pressure
Binge Drinking	−0.18 **	−0.24 **	−0.45 ***

** *p* < *0*.01, *** *p* < *0*.001.

**Table 4:** Regression analysis of DRSE on binge drinking.

Gender	Collinearity							
		B	*SD*	*β*	*t*	*p*	T	VIF
Male	Social Pressure	−0.14	0.02	−0.42	−6.54	0.001	0.93	1.07
	Opportunistic Drinking	−0.07	0.02	−0.20	−3.08	0.002	0.94	1.07
	*R*^2^ = 0.23, adj *R*^2^ = 0.22, *F* = 33.49, sig. = 0.001, DW = 1.47							
Female	Social Pressure	−15	0.03	−0.40	−5.49	0.001	1	1
	*R*^2^ = 0.16, adj *R*^2^ = 0.15, *F* = 30.11, sig. = 0.001, DW = 1.61							

B = unstandardised regression coefficients; β = standardised regression coefficient; VIF = Variance Inflation Factor.
